# Supplementary material for: Are long telomeres better than short? Relative contributions of genetically predicted telomere length to neoplastic and non-neoplastic disease risk and population health burden
Source: PLoS One. 2020 Oct 8;15(10):e0240185. doi: 10.1371/journal.pone.0240185 (PMC7544094; doi:10.1371/journal.pone.0240185)
Supplement: S1 File — (PDF) [file pone.0240185.s001.pdf]

SUPPLEMENTAL METHODS:

Burden of Disease Attributable to Telomere Length

To estimate the population-level disease burden associated with telomere length, we multiplied the odds ratios calculated by Haycock et al. (2017) by the corresponding Disability-Adjusted Life Years (DALY) metric published by the Institute of Health Metrics and Evaluation (IHME) at the University of Washington. DALYs can be thought of as years of 'healthy life' lost due to disease, and are calculated as the sum of two measures: Years of Life Lost (YLL), which reflects premature mortality attributable to disease, and Years Lost due to Disability (YLD), which reflects the burden of the disease experienced by the patient prior to death. Unlike prevalence, incidence, and mortality, DALYs allow for the direct quantitative comparison of diseases with vastly different trajectories. For instance, the burden of non-fatal chronic diseases such as cardiovascular disease can be directly compared to that of rapidly fatal diseases such as aggressive cancer.

Calculating Excess Disease Burden Associated with gTL

All DALY data comes from the IHME, and was downloaded through the GBD Results Tool (http://ghdx.healthdata.org/gbd-results-tool) in July 2019. Age-standardized DALY data for the 2017 year were downloaded for the population of Europe. Europe was chosen to maximize consistency with the Haycock et al. meta-analysis, where all participants were of European ancestry.

The odds ratios in Haycock et al. are given relative to longer genetically predicted telomere length. This makes comparing the ORs of neoplastic and degenerative diseases challenging, because while a 5-fold increase in cancer risk associated with longer gTL would have an OR of 5.0, a 5-fold decrease in degenerative disease risk would have an OR of 0.2. As a result, the impact of gTL on cancer risk seems initially much greater than the impact on degenerative disease risk. In order to make the comparison between neoplastic and non-neoplastic disease intuitive, we present the Haycock et al. ORs relative to the hypothesized gTL 'effect.' In other words, ORs for neoplastic diseases are given relative to longer gTL, while ORs or non-neoplastic diseases are given relative to shorter gTL. The ORs for non-neoplastic diseases were converted by taking their inverse (e.g., and OR of 0.20 associated with longer gTL becomes 5.0 associated with shorter gTL).

To capture the excess DALYs per 100,000 persons attributable to genetically predicted telomere length, we used the formula "(OR-1) x DALY". In other words, if an OR=1 reflects baseline odds of disease, we subtract OR-1 to capture odds of disease predicted by TL in excess of baseline. This value is multiplied by the corresponding DALY to estimate the excess disease burden experienced by the European population due to long telomere length.

95% confidence intervals for the measure of excess DALYs is reported. It is calculated by the same equations described above.

Calculating Excess Disease Incidence Associated with gTL

While the DALY measure is best suited for comparing the burden of diseases with vastly different trajectories, we were also interested in exploring the contribution of gTL to simple disease incidence. Incidence data for the European population were similarly downloaded from the IHME database, using 2017 estimates. Calculations of excess incidence associated with gTL were identical to those used for burden.

Weighting of IHME Incidence and DALY Data

In some cases, Haycock et al. report odds ratios for narrower disease definitions than the available IHME data. For instance, there is a single IHME estimate for cancers of the trachea, bronchus, and lungs, but there are two available odds ratios: squamous cell carcinoma of the lung and adenocarcinoma of the lung. We used data from the United States SEER cancer registry database to calculate weighting factors, and thereby interpolated the DALYs and incidence for each cancer defined by Haycock et al.

Weighting factors were calculated by dividing the number of cases of each cancer type included by Haycock (e.g., ‘lung adenocarcinoma’) by the total number of cases in the broader cancer grouping defined by the IHME (e.g., ‘lung and bronchus cancers’). Efforts were made to specify search criteria in SEER to most closely match both the Haycock and IHME disease definitions. Specific SEER search criteria for each cancer type are described below.

Prevalence and Incidence Data – SEER and Literature Review

For all cancers, case counts were determined from SEER, a database of United States cancer records made publicly available by the National Cancer Institutes.

A single database within SEER was used to determine incidence of all cancers: *“Incidence – SEER 18 Regs Research Data + Hurricane Katrina Impacted Louisiana Cases, Nov 2018 Sub (2000-2016) <Katrina/Rita Population Adjustment>”*

The “Rate” module was used to obtain case counts of all cancers within the 2000-2016 database consisting of 18 cancer registries across the US.

Cases were restricted to individuals of “white” race, in an effort to maintain consistency with ORs derived from participants of European ancestry in the Haycock et al. GWAS meta-analysis. Efforts were made to identify a comparable European database of cancers, but the identified resources did not provide the histological diagnoses necessary for weighting.

Trachea, Bronchus, and Lung Cancers:

- Haycock et al. provides ORs for adenocarcinoma and squamous cell carcinoma. Only the OR for adenocarcinoma is statistically significant, and therefore is included in this analysis. The IHME provides data only for a broad grouping of cancers defined as ‘Trachea, bronchus, and lung cancers.’ Review of the associated ICD-10 codes provided by IHME confirms this definition, with the exception of code D02.3, which includes ‘carcinoma in situ of other parts of respiratory system.’ As other parts of respiratory system were not included elsewhere (e.g., C30-C32 not included), they were excluded in SEER search. Additionally, review of ICD10 codes reveals that the

IHME includes cancers in situ, benign neoplasms, and neoplasms of unknown behavior.

- The SEER search for total number of ‘trachea, bronchus, and lung cancers’ was defined as follows:
    - {Site and Morphology.Site recode ICD-O-3/WHO 2008} = 'Lung and Bronchus', ‘Trachea, Mediastinum, and Other Respiratory Organs’ AND {Site and Morphology.Primary Site – labeled} = ‘C33.9-Trachea’,C34.1-Upper lobe,lung’,C34.2-Middle lobe,lung’,C34.3-Lower lobe,lung’,’C34.8-Overlapping lesion of lung’,’C34.9-Lung,NOS.’
    - The search yielded 720,315 total cases of cancers consistent with the IHME criteria. (If restricted to malignant only, this same search yields 719,176 cases.)
- The SEER search for number of adenocarcinomas of the lung was defined as follows:
    - {Site and Morphology.Site recode ICD-O-3/WHO 2008} = 'Lung and Bronchus' AND {Site and Morphology.Histology recode - broad groupings} = '8140-8389: adenomas and adenocarcinomas' AND {Site and Morphology.Primary Site – labeled} =’C34.1-Upper lobe,lung’,C34.2-Middle lobe,lung’,C34.3-Lower lobe,lung’,’C34.8-Overlapping lesion of lung’,’C34.9-Lung,NOS.’ This search was restricted to malignant cancers only.
    - This search yielded 255,644 cases of adenocarcinoma of the lung.
    - Several other search methods were attempted, including one without the criterion for ‘primary site.’ This search yielded slightly more cases, at 262,538. However, review of the ICD-O-3 codes raised concern that this number included metastatic cancers to the lung.
- The weighting factor for adenocarcinoma of the lung was therefore 262,538/720,315 = 0.35. In other words, 35% of all trachea, bronchus, and lung cancers are adenocarcinomas of the lung. Accordingly, the Incidence and DALY values extracted from the IHME database were multiplied by 0.35 to approximate the respective values for adenocarcinoma.

Ovarian Cancer:

- Haycock et al. provides five odds ratios for ovarian cancers: serous LMP, serous invasive, clear cell, endometrioid, and mucinous. There are multiple ICD-O-3 codes corresponding to each of these, but the SEER case definitions do not provide broad groupings for these cancers, as they do for the lung cancers. Instead, all cases of ovarian cancer exhibiting malignant behavior were selected using the following selection criteria: “{Site and Morphology.Site recode ICD-O-3/WHO 2008} = 'Ovary'.” The output was stratified by ICD-O-3 Histology/behavior codes and searched for the terms “serous,” “clear cell,” “endometrioid,” and “mucin.” As only the OR for serous LMP was statistically significant, the associated ICD-O-3 codes were selected and used for a targeted SEER search for Serous LMP cases only.
- “Serous LMP” refers to tumors of “low malignant potential.” These cases are not captured by selection criteria that restrict to cases of malignant behavior, and therefore this was not used as a criterion. Serous LMP cancers typically refer to those of “borderline malignancy,” but so as to avoid underestimating the weighting factor,

we also included “in situ” neoplasms. This added only an additional 30 cases (572 vs 542).

  - 8441/2: Serous cystadenocarcinoma, NOS, in situ
  - 8442/1: Serous cystadenoma, **borderline malignancy**
  - 8442/2: Proliferating serous carcinoma in situ
  - 8460/2: Papillary serous cystadenocarcinoma, NOS, in situ
  - 8461/2: Serous surface papillary carcinoma, in situ
  - 8462/1: Serous papillary cystic tumor of **borderline malignancy**
  - 8462/2: Serous papillary cystadenocarcinoma in situ
  - 9014/2: Serous adenocarcinofibroma, in situ

- The ICD-O-3 codes included in each cancer definition are outlined below. A SEER training module on Epithelial Ovarian Cancers was used as reference to understand cancer case definitions. The results of the SEER search are believed to be broadly consistent with the information presented in the training module: https://training.seer.cancer.gov/ovarian/abstract-code-stage/ovarian-morphology.html
- To calculate the total number of ovarian cancer cases, the “{Site and Morphology.Site recode ICD-O-3/WHO 2008} = 'Ovary'.” criterion was used, not restricted to malignant cases. It yielded 83,813 total cases. Adding a primary site criterion yielded the same result.
- The weighting factor for serous LMP was therefore defined as 572/83,813 = 0.00682.

Brain and Nervous System Cancers:

- The IHME provides a single incidence and DALY data point for ‘brain and nervous system cancers,’ defined as ICD-10 codes C70-72.9, referring to malignant neoplasms of the meninges, brain, spinal cord, cranial nerves, and other parts of the CNS. As these are limited to malignant neoplasms, “malignancy” was used as a criterion in the SEER search for total number of brain and CNS cancer cases. The search criteria were defined as follows:
  - {Site and Morphology.Site recode ICD-O-3/WHO 2008} = 'Brain and Other AND Site and Morphology.Primary Site – labeled} =’C70.0-Cerebral mininges’,C70.1-Spinal meninges’,C70.9-Meninges,NOS’,C71.0-Cerebrum’,C71.1-Frontal lobe’,’C71.2-Temporal lobe’,’C71.3Parietal lobe’,’C71.4-Occipital lobe’,’C71.5-Ventricle’,’C71.6-Cerebellum’,’C71.7-Brain stem’,’C71.8-Overlapping lesion of brain’,’C71.9-Brain,NOS’,’C72.0-Spinal cord’,’C72.1-Cauda equina’,’C72.2-Olfactory nerve’,’C72.3-Optic nerve’,’C72.4-Acoustic nerve’,’C72.5-Cranial nerve,NOS’,’C72.8-Overlapping lesion of brain & CNS’,’C72.9-Nervous system,NOS’
  - Note that excluding the primary site criterion did not alter the case number. The search yielded 81,431 cases.
- Haycock provides two ORs of interest: glioma and neuroblastoma.
- Neuroblastoma was defined as {Site and Morphology.Site recode ICD-O-3/WHO 2008} = 'Brain and Other AND {Site and Morphology.ICD-O-3 Hist/behave }= '9500/3: Neuroblastoma, NOS'. This search yielded 103 cases. This did not include an additional 24 cases of ganglioneuroblastoma, and 3 cases of olfactory neuroblastoma. The weighting factor was defined as 103/81,431 = 0.001265.

- Glioma was challenging to define. The publications cited by Haycock et al. as data sources were reviewed. It was noted that the majority of patients included in these trials had glioblastoma (see Wrensch et al and Walsh et al.). The Wrensch et al publication also states that the trial contributing the majority of patients – the AGS trial – defined its inclusion criteria as a histologically confirmed incident glioma with ICD-O morphology codes 9380-9481 (but last available ICD-O-3 is 9474/3). These same codes were used to define the SEER search for gliomas. The search criteria were as follows:
    - o {Site and Morphology.Site recode ICD-O-3/WHO 2008} = 'Brain and Other Nervous System' AND {Site and Morphology.ICD-O-3 Hist/behav, malignant} = '9380/3: Glioma, malignant','9381/3: Gliomatosis cerebri','9382/3: Mixed glioma','9383/3: Subependymoma, malignant','9384/3: Subependymal giant cell astrocytoma, malignant','9390/3: Choroid plexus papilloma, malignant','9391/3: Ependymoma, NOS','9392/3: Ependymoma, anaplastic','9393/3: Papillary ependymoma, NOS','9394/3: Myxopapillary ependymoma, malignant','9400/3: Astrocytoma, NOS','9401/3: Astrocytoma, anaplastic','9410/3: Protoplasmic astrocytoma','9411/3: Gemistocytic astrocytoma','9413/3: Dysembryoplastic neuroepithelial tumor, malignant','9420/3: Fibrillary astrocytoma','9421/3: Pilocytic astrocytoma, malignant','9423/3: Polar spongioblastoma','9424/3: Pleomorphic xanthoastrocytoma','9430/3: Astroblastoma','9440/3: Glioblastoma, NOS','9441/3: Giant cell glioblastoma','9442/3: Gliosarcoma','9444/3','9450/3: Oligodendroglioma, NOS','9451/3: Oligodendroglioma, anaplastic','9460/3: Oligodendroblastoma','9470/3: Medulloblastoma, NOS','9471/3: Desmoplastic nodular medulloblastoma','9472/3: Medullomyoblastoma','9473/3: Primitive neuroectodermal tumor','9474/3: Large cell medulloblastoma'
    - o This search yields 74,973 cases of glioma. The weighting factor for gliomas is therefore defined as 74,973/81,431 = 0.92
-

ICD-10 Codes Mapped to IHME Disease Definitions
To ensure diagnoses were being appropriately grouped, the ICD-10 codes attributed to each DALY disease category were reviewed. This documentation, published by the Institute of Health Metrics and Evaluation (IHME) is freely available here: http://ghdx.healthdata.org/record/global-burden-disease-study-2016-gbd-2016-causes-death-and-nonfatal-causes-mapped-icd-codes

Conditions Excluded Because There Is No Available DALY
The following conditions are excluded from our analysis despite having statistically significant ORs in the Haycock et al. analysis, because IHME data is not available.
- Abdominal aortic aneurysm
- Celiac Disease

The following conditions from had significant ORs in the Li et al. analysis but were also excluded because IHME data is not available.
- Hypothyroidism
- Uterine polyps

SNPs Associated with Telomere Length:

A list of all SNPs reported to be associated with telomere length in the published literature was compiled as follows: 1) A Pubmed search for the term "telomere length" AND (SNP OR "polymorphism, single nucleotide") AND human was completed on November 26, 2019, yielding 211 results. All abstracts were reviewed, and studies that included a direct measure of association between LTL and SNPs were selected. Studies reporting associations w/ TL in other tissues (e.g., buccal tissue) were excluded. Inclusion was not limited to specific statistical methods, and the list includes GWAS as well as hypothesis-driven tests of association. Only studies measuring associations in cancer-free participants are included. 2) An additional 2 studies were identified in the GWAS Catalog in November 2019. 3) All 16 SNPs reported by Haycock et al. (2017) were included, as well as any other significant SNPs in the original publications used for meta-analysis. These were captured by the above literature search methods.

Chromosomal position data for all SNPs was drawn from the PubMed SNP database, using assembly GRCh38.p7. Effect alleles are reported as in their original publications. 'Other alleles' are reported as in the publication where available, or otherwise as reported in the PubMed SNP database. Effect allele frequencies are based on the '1000Genomes' global population data, found in the PubMed SNP database.

Linkage Disequilibrium Among SNPs Associated with Telomere Length

Linkage disequilibrium (LD) between SNPs on the same chromosome is given as R_2 values, obtained from the 'LDlink' suite of applications, provided by National Cancer Institute Division of Cancer Epidemiology & Genetics (https://ldlink.nci.nih.gov). The 'LDmatrix' tool was used to generate tables of R_2 values for all chromosomes with at least two identified SNPs. While there are a number of exceptions, the majority of GWAS studies listed in Table 1 were based on European populations. In order to maximize consistency with GWAS data, LD was based on five European populations: Utah Residents from North and West Europe, Toscani in Italia, Finnish in Finland, British in England and Scotland, and Iberian population in Spain. R_2 values were visualized in 'R' with the 'corrplot' function. Figure # includes all chromosomes where at least two SNPs were in LD, based on a cutoff of R_2=0.50. Although there is no LD in chromosome 20, it was also included to highlight the finding that there is are three SNPs within the RTEL1 gene that not in LD with one another.

TelNet Gene Function Table

The function of genes implicated in telomere length by GWAS studies was explored through the TelNet Database in December 2018 in order to identify possible mechanism of telomere length regulation (https://malone2.bioquant.uni-heidelberg.de/fmi/webd/TelNet). Where no known function in or association with telomere biology is known, rows remain blank.
