# Supplementary material for: Are long telomeres better than short? Relative contributions of genetically predicted telomere length to neoplastic and non-neoplastic disease risk and population health burden
Source: PLoS One. 2020 Oct 8;15(10):e0240185. doi: 10.1371/journal.pone.0240185 (PMC7544094; doi:10.1371/journal.pone.0240185)
Supplement: Attachment — Submitted filename: PlosOne, Telomeres.docx [file pone.0240185.s002.docx]

Protsenko et al have collaborated on an elegant study that adds new insight into our understanding of the potential role of the inter-individual telomere length (TL) variation in human health. I commend them for the breath of their findings. Below are minor comments.

Introduction

L81-84. “These estimates from family-based studies…”: While I agree with the statement, robust associations of leukocyte TL (LTL) were observed between newborns and their parents. One might argue that a shared intrauterine environment contributes to these associations. Still, the impact of the environment on LTL, in my view, is much less than that of the genetic makeup of the individual.

L 87-89. “A recent study using genome-wide complex trait analysis…”: Statement is based on a study that used salivary DNA TL measurements by qPCR. Findings are less robust than those based on larger datasets generated using LTL measured by either Southern blotting or qPCR.

L 108-115. “It has been proposed that the opposing directions…”: Statements are imprecise and should be rewritten. Ref 53 discusses the role of evolutionary forces, which by definition work principally during the reproductive years, in fashioning an *optimal* TL in humans. It underscores that in contemporary humans, the lasting impact of these forces is expressed during the post-reproductive years. The selective evolutionary forces on TL is also implicit in the narrative of ref 54 and PMID 26936823. Moreover, other publications further elaborate on this idea (PMID 30631124, PMID: 32427393).

Discussion

L239-242. When we apply our method to estimate the excess incidence of these conditions, we find that…”: Indeed, this is the key point of the paper, yet it is not emphasized in the abstract.

L347-349. “Second, as environmental factors can influence telomere length throughout the whole lifespan…”: No doubt, this statement is true, particularly during infancy/early childhood, when TL shortening is rapid, but the overall magnitude of the effect is minor compared to the inter-individual TL variation at birth. This is barely appreciated from studies that generate T/S data by qPCR.
