# Supplementary material for: Are long telomeres better than short? Relative contributions of genetically predicted telomere length to neoplastic and non-neoplastic disease risk and population health burden
Source: PLoS One. 2020 Oct 8;15(10):e0240185. doi: 10.1371/journal.pone.0240185 (PMC7544094; doi:10.1371/journal.pone.0240185)
Supplement: Attachment — Submitted filename: Protsenko Author response.docx [file pone.0240185.s003.docx]

Dear Drs. Saretzki and Aviv,

We thank you for your insightful and constructive comments on our manuscript titled “Are Long Telomeres Better Than Short?: Relative Contributions of Genetically Predicted Telomere Length to Neoplastic and Non-Neoplastic Disease Risk and Population Health Burden”. We have carefully reviewed all the comments and addressed each of them either by revising the manuscript accordingly or providing explanations and clarifications. Please see attached the revised manuscript and the response letter. Thank you again for the opportunity to submit our manuscript to your journal and we look forward to hearing back from you.

Sincerely,

Jue Lin, Ph. D.
Department of Biochemistry and Biophysics

Room S316, 600 16th Street

San Francisco, CA 94158

Phone (415) 476-7284

Email: jue.lin@ucsf.edu

Response to reviewer’s comments

Introduction

-L81-84. “These estimates from family-based studies…”: While I agree with the statement, robust associations of leukocyte TL (LTL) were observed between newborns and their parents. One might argue that a shared intrauterine environment contributes to these associations. Still, the impact of the environment on LTL, in my view, is much less than that of the genetic makeup of the individual.

**Authors’ response**: We have revised this sentence to reflect the contribution of a shared intrauterine environment. The new version now reads as follows:

“These estimates from family-based studies reflect both the genetic contribution and shared environmental factors due to the relatedness in the study participants, as well as a shared intrauterine environment in some cases, and therefore are likely to be higher than the true portion of telomere length variation determined by genetic inheritance”.

-L 87-89. “A recent study using genome-wide complex trait analysis…”: Statement is based on a study that used salivary DNA TL measurements by qPCR. Findings are less robust than those based on larger datasets generated using LTL measured by either Southern blotting or qPCR.

**Authors’ response:** We agree with Dr. Aviv that TL results using salivary DNA are more likely impacted by preanalytical factors, therefore less robust. We have added the following sentence in the revised version.

“However, it should be noted that this study used salivary DNA for TL measurements, which are less robust than DNA from leukocytes.”

-L 108-115. “It has been proposed that the opposing directions…”: Statements are imprecise and should be rewritten. Ref 53 discusses the role of evolutionary forces, which by definition work principally during the reproductive years, in fashioning an *optimal* TL in humans. It underscores that in contemporary humans, the lasting impact of these forces is expressed during the post-reproductive years. The selective evolutionary forces on TL is also implicit in the narrative of ref 54 and PMID 26936823. Moreover, other publications further elaborate on this idea (PMID 30631124, PMID: 32427393).

**Authors’ response:** We thank Dr. Aviv for this very helpful suggestion. We have revised the statement and it now reads as follows:

“It has been proposed that the opposing directions of these associations imply that human telomere length has evolved to balance the disease risks imposed by both short and long telomeres[53, 54], and thereby achieving an optimal length over successive generations. It would seem that this argument fails to consider the fact that the manifestation of the majority of the diseases associated with long or short telomeres, with the exception of a number of rare cancers (e. g. neuroblastoma and testicular cancer, some types of leukemia and lymphoma and type I diabetes mellitus), happens in later life, well past the reproductive period to have real evolutional pressure on the population. However, this finding among contemporary humans could alternatively suggest that selection forces have acted on telomere length precisely to lessen the impact of degenerative and neoplastic diseases during the reproductive years [55, 56].”

Discussion

-L239-242. When we apply our method to estimate the excess incidence of these conditions, we find that…”: Indeed, this is the key point of the paper, yet it is not emphasized in the abstract.

**Authors’ response:** We revised the abstract to emphasize this point. The revised Results section of the abstract is shown below:

**“Results**: Our analysis shows that, despite the markedly larger ORs of neoplastic disease, the large incidence of degenerative diseases causes the excess incidence attributable to gTL to balance that of neoplastic diseases.”

-L347-349. “Second, as environmental factors can influence telomere length throughout the whole lifespan…”: No doubt, this statement is true, particularly during infancy/early childhood, when TL shortening is rapid, but the overall magnitude of the effect is minor compared to the inter-individual TL variation at birth. This is barely appreciated from studies that generate T/S data by qPCR.

**Authors’ response:** We **agree with** Dr. Aviv that the magnitude of TL change over adult life is much smaller compared to the inter-person variation at birth. We have added a sentence to reflect this (see below).

“Second, as environmental factors can influence telomere length throughout the whole lifespan, and perhaps differentially during different developmental stages (childhood, adulthood and geriatric), the disease risks caused by short or long telomere length can change accordingly. Although it should be noted that compared to the inter-individual TL variation at birth, the overall magnitude of the effect of environmental factors is smaller [69].”
